# Comparison of overall survival on surgical resection versus transarterial chemoembolization with or without radiofrequency ablation in intermediate stage hepatocellular carcinoma: a propensity score matching analysis

**DOI:** 10.1186/s12876-020-01235-w

**Published:** 2020-04-10

**Authors:** Chih-Wen Lin, Yaw-Sen Chen, Gin-Ho Lo, Yao-Chun Hsu, Chia-Chang Hsu, Tsung-Chin Wu, Jen-Hao Yeh, Pojen Hsiao, Pei-Min Hsieh, Hung-Yu Lin, Chih-Wen Shu, Chao-Ming Hung

**Affiliations:** 1 Division of Gastroenterology and Hepatology, E-Da Dachang Hospital, I-Shou University, Kaohsiung, Taiwan; 2 Division of Gastroenterology and Hepatology, Department of Medicine, E-Da Hospital, I-Shou University, Kaohsiung, Taiwan; 3 Health Examination Center, E-Da Hospital, I-Shou University, Kaohsiung, Taiwan; 4 School of Medicine, College of Medicine, I-Shou University, 82445, No. 1, Yida Road, Jiaosu Village, Yanchao District, Kaohsiung, Taiwan; 5 Graduate Institute of Medicine, College of Medicine, Kaohsiung Medical University, Kaohsiung, Taiwan; 6 School of Chinese Medicine, College of Chinese Medicine, China Medical University, Taichung, Taiwan; 7 Research Center for Traditional Chinese Medicine, China Medical University Hospital, Taichung, Taiwan; 8 Department of Surgery, E-Da Hospital, I-Shou University, No. 1, Yida Road, Jiaosu Village, Yanchao District, Kaohsiung, 82445 Taiwan; 9 Department of Surgery, E-Da Cancer Hospital, I-Shou University, Kaohsiung, Taiwan

**Keywords:** Hepatocellular carcinoma, Barcelona clinic liver Cancer stage B, Overall survival, Surgical resection, Transcatheter arterial chemoembolization, Radiofrequency ablation

## Abstract

**Background:**

Patients with Barcelona Clinic Liver Cancer (BCLC) stage B hepatocellular carcinoma (HCC) are recommended to undergo transcatheter arterial chemoembolization (TACE). However, TACE in combination with radiofrequency ablation (RFA) is not inferior to surgical resection (SR), and the benefits of surgical resection (SR) for BCLC stage B HCC remain unclear. Hence, this study aims to compare the impact of SR, TACE+RFA, and TACE on analyzing overall survival (OS) in BCLC stage B HCC.

**Methods:**

Overall, 428 HCC patients were included in BCLC stage B, and their clinical data and OS were recorded. OS was analyzed by the Kaplan-Meier method and Cox regression analysis.

**Results:**

One hundred forty (32.7%) patients received SR, 57 (13.3%) received TACE+RFA, and 231 (53.9%) received TACE. The OS was significantly higher in the SR group than that in the TACE+RFA group [hazard ratio (HR): 1.78; 95% confidence incidence (CI): 1.15–2.75, *p* = 0.009]. The OS was significantly higher in the SR group than that in the TACE group (HR: 3.17; 95% CI: 2.31–4.36, *p* < 0.0001). Moreover, the OS was significantly higher in the TACE+RFA group than that in the TACE group (HR: 1.82; 95% CI: 1.21–2.74, *p* = 0.004). The cumulative OS rates at 1, 3 and 5 years in the SR, TACE+RFA, and TACE groups were 89.2, 69.4 and 61.2%, 86.0, 57.9 and 38.2%, and 69.5, 37.0 and 15.2%, respectively. After propensity score matching, the SR group still had a higher OS than those of the TACE+RFA and TACE groups. The TACE+RFA group had a higher OS than that of the TACE group.

**Conclusion:**

The SR group had higher OS than the TACE+RFA and TACE groups in BCLC stage B HCC. Furthermore, the TACE+RFA group had higher OS than the TACE group.

## Background

Hepatocellular carcinoma (HCC) is the fifth most common cancer but the third most lethal cancer worldwide [1]. The Barcelona Clinic Liver Cancer (BCLC) system is widely utilized in the American Association for the Study of Liver Disease (AASLD), European Association for the Study of Liver (EASL) and Asian-Pacific Associated for the Study of the Liver (APASL) guidelines for the treatment of HCC [2–4]. Patients with stage B (intermediate stage) HCC are recommended to undergo transcatheter arterial chemoembolization (TACE) based on the BCLC system [2–4]. However, surgical resection (SR) and radiofrequency ablation (RFA) are curative therapies in BCLC stage 0/A and are alternative therapies for selected patients with BCLC stage B in clinical practice [5–7]. Previous studies have shown that TACE combined with RFA (TACE+RFA) has a better overall survival (OS) than TACE in BCLC stage B [6, 8, 9]. Moreover, some studies have shown that SR can have a better OS than TACE with or without RFA in BCLC stage B [5–7]. However, TACE+RFA is not inferior to SR for patients with HCC within the Milan criteria [10]. Furthermore, TACE + RFA is not inferior to SR for patients with HCC within BCLC stage A or B after propensity score-based analysis [6]. Hence, this study aims to compare the impact of SR, TACE + RFA, and TACE on the OS of HCC patients with BCLC stage B. Each patient was treated with one of these three therapies. Furthermore, we compared the OS of patients in each group using propensity score matching (PSM) to minimize potential bias in the results.

## Methods

### Patients and follow-up

**Fig. 1:**
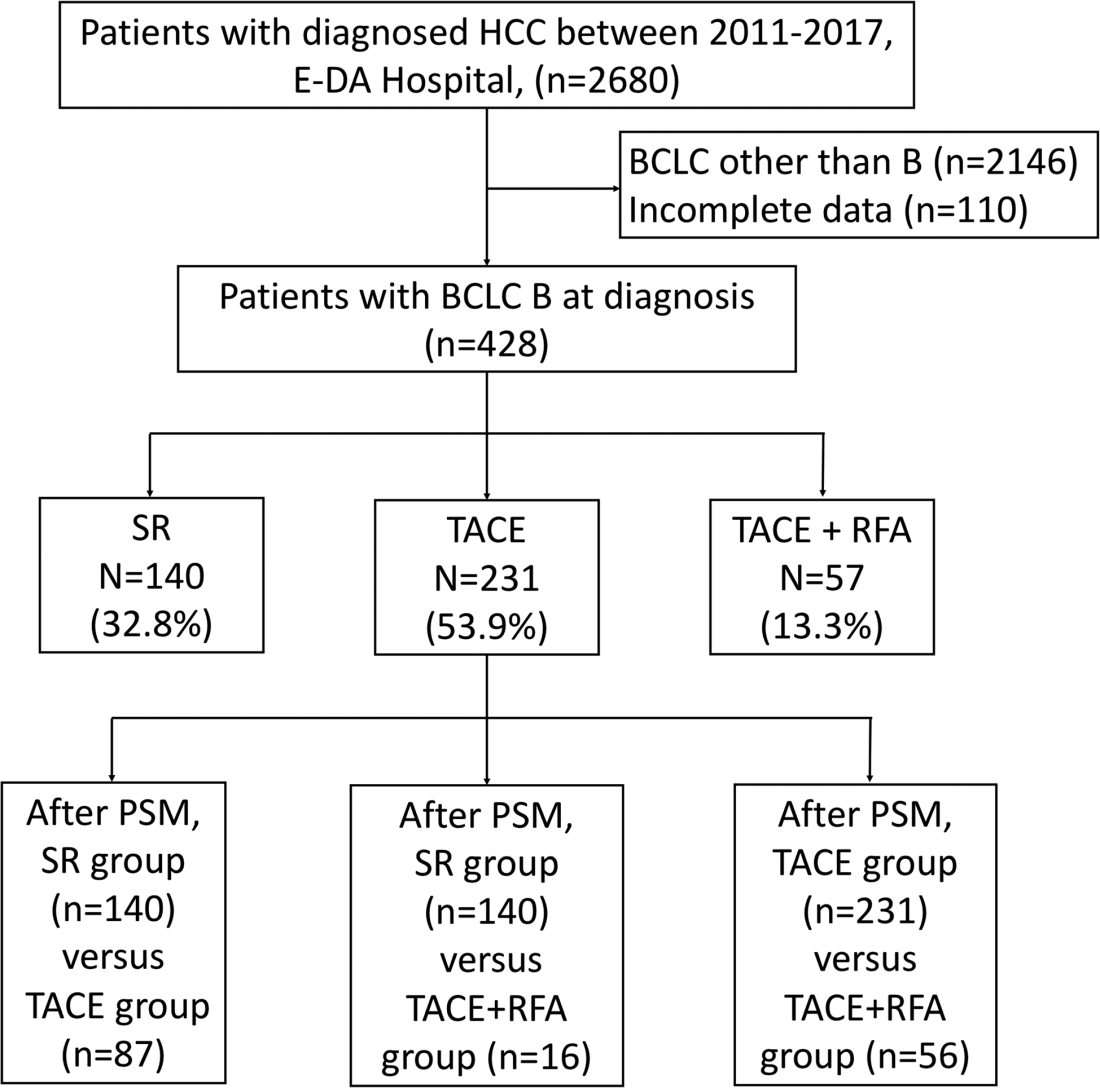
Study flowchart and inclusion of participants

We retrospectively collected information on 2680 patients diagnosed with HCC between 2011 and 2018 at E-Da Hospital, I-Shou University, Kaohsiung, Taiwan. Two thousand and one hundred forty-six patients were excluded due to BCLC stage 0, A, C, and D, and 110 patients had incomplete data in BCLC stage B. Finally, 428 patients with BCLC stage B were included in this retrospective study (Fig. 1). The study was conducted in accordance with the guidelines of the International Conference on Harmonization for Good Clinical Practice and was approved by the Ethics Committee of E-Da Hospital, I-Shou University (EMRP-107-130). Patients were diagnosed with HCC based on histological confirmation or at least one typical imaging method according to the recommendations of the AASLD [2]. Clinicopathological parameters, including demographic data, smoking, excessive alcohol use, hepatitis status, serum total bilirubin, international normalization ratio (INR), liver cirrhosis, Child-Pugh (CP) class, tumor size, tumor number, alpha-fetoprotein (AFP), mortality, and follow-up time, were examined. Tumor number and tumor size were mostly determined based on radiologic findings and confirmed by pathologic findings if appropriate. Liver cirrhosis was diagnosed based on pathologic findings and/or evaluated by ultrasound, computed tomography (CT), or magnetic resonance imaging (MRI). The functional status of the liver was evaluated using the CP scoring system.

Patients were treated with SR, TACE+RFA, and TACE, and our multidisciplinary team chose suitable therapy. The criteria for SR were resectable tumors, sufficient residual liver volume, CP class A or selected CP class B patients, or absence of ascites and hypersplenism. The indications of TACE+RFA were CP class A or B and absent ascites. The indications of TACE were CP class A or B and absent massive ascites. Patients were divided into the SR group, TACE+RFA group, and TACE group.

Patients were followed up every three to 6 months by abdominal ultrasound, CT or MRI and AFP. OS was defined as the time from the date of diagnosis to the date of death or last visit, and the last follow-up time was June 2019.

### Data analysis and statistics

All statistical analyses were performed using SPSS ver. 23.0 (SPSS, Chicago, IL, USA). Numerical data were expressed as medians and ranges. Categorical data were described using numbers and percentages. OS was determined using the Kaplan-Meier method and compared with patients receiving different treatments. Cox proportional hazards regression analysis of OS in HCC patients was performed according to different treatments. Moreover, we used logistic regression to perform PSM with sex, age, cirrhosis, CP class, tumor size, and tumor number for patients to reduce bias in our analyses. Each treatment group was matched with the control group (SR group or TACE group) according to the generated PSM using a caliper width of 0.2. On the completion of matching, the baseline covariates were compared using the paired t-test or Mann–Whitney U test for continuous variables and the chi-square test for categorical variables. A *p*-value < 0.05 was used to determine statistical significance.

## Results

### Baseline demographic data before propensity score matching

A total of 428 HCC patients were included in this study (Fig. 1). The demographic and clinical features of the 428 patients (77.8% male, median age of 63 years) are shown in Table 1. Regarding the etiology of HCC, 47.9% of the patients had HBV infection, 32.4% had HCV infection, and 42.3% had excessive alcohol use. Approximately 54.7% of patients had liver cirrhosis, and of those patients, 86.9% had CP class A disease. Many (67.5%) of the patients had tumors ≥5 cm in size, and 65.0% of the patients had multiple tumors.

**Table 1:** Basic demographic data of patients with BCLC stage B hepatocellular carcinoma of various treatments

Variable	SR (*n* = 140)	TACE (*n* = 231)	TACE+RFA (*n* = 57)	Total (*n* = 428)	*P*-value
Male	117 (83.6)	173 (74.9)	43 (75.4)	333 (77.8)	0.134
Age (years)	62 (35–82)	64 (29–91)	64 (28–86)	63 (25–91)	0.311
Smoking	68 (48.6)	113 (48.9)	27 (47.4)	208 (48.6)	0.978
Alcohol use	58 (41.4)	100 (43.3)	23 (40.4)	181 (42.3)	0.894
HBV positive	70 (50.0)	103 (44.6)	32 (56.1)	205 (47.9)	0.245
HCV positive	30 (21.4)	90 (39.0)	21 (36.8)	141 (32.9)	0.002
Total Bilirubin	1.03 ± 0.43	1.34 ± 1.14	1.40 ± 0.66	1.24 ± 0.91	0.003
INR	1.00 ± 0.06	1.06 ± 0.12	1.10 ± 0.14	1.05 ± 0.11	< 0.0001
Cirrhosis	36 (25.7)	155 (67.1)	43 (75.4)	234 (54.7)	< 0.0001
Child-Pugh class A	134 (95.7)	194 (84.0)	44 (77.2)	372 (86.9)	< 0.0001
Tumor size	8.2 ± 3.3	7.0 ± 3.8	5.5 ± 2.6	7.0 ± 3.6	0.001
Tumor size≥5 cm	127 (90.7)	149 (64.5)	25 (43.8)	289 (67.5)	< 0.0001
Tumor number (≥3)	49 (35.0)	178 (77.1)	51 (89.5)	278 (65.0)	< 0.0001
AFP (ng/mL) ≥ 200	34 (24.3)	50 (21.6)	7 (12.1)	91(21.3)	0.171
Mortality	50 (35.7)	173 (74..9)	34 (59.6)	257 (60.0)	< 0.0001
Follow-up times (months)	39 (1–98)	22 (1–97)	37 (3–95)	29 (1–98)	< 0.001

*BCLC stage* Barcelona clinic liver cancer; *SR* Surgical resection; *TACE* Transcatheter arterial chemoembolization; *RFA* Radiofrequency ablation; *HBV* Hepatitis B virus; *HCV* Hepatitis C virus; *AFP: INR* International normalize ratio; Alpha-fetoprotein;

### Overall survival of patients in the total and different treatment groups

**Fig. 2:**
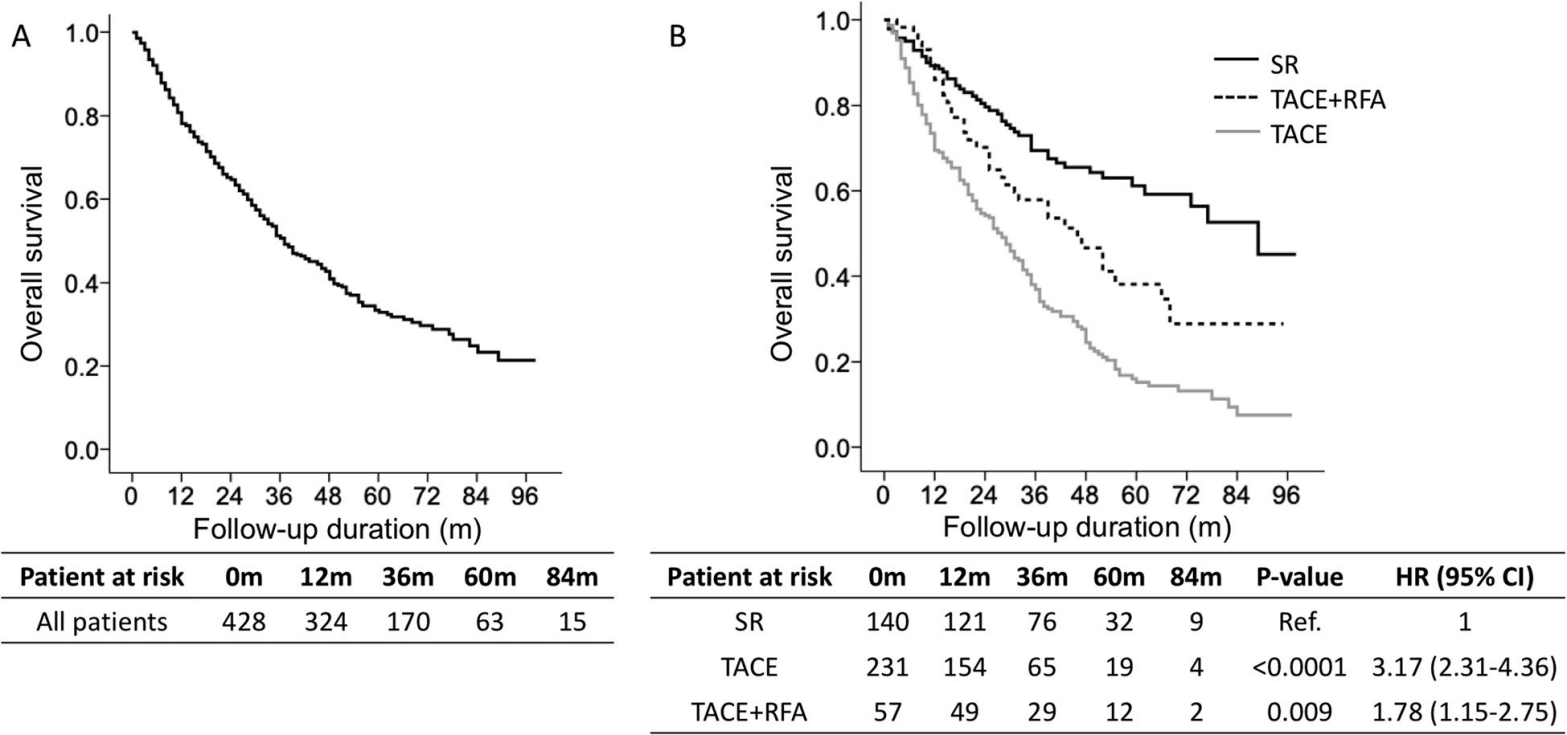
Overall survival in Barcelona Clinic Liver Cancer stage B hepatocellular carcinoma (HCC) patients. Overall survival in all 428 HCC patients (**a**). Overall survival based on Cox regression analysis in HCC patients with different treatments before propensity score matching (**b**)

Of the 428 patients, 257 (60.0%) died, and the median follow-up duration was 29 (range, 1–98) months (Table 1). The mortality rate was 24.8% per person-year. The cumulative OS rates at 1, 3, and 5 years were 80.8, 50.6 and 32.8%, respectively (Fig. 2a). Among the 428 patients, 140 (32.7%) patients received SR, 231 (53.9%) received TACE+RFA, and 57 (13.3%) received TACE (Table 1). The OS was significantly better in the SR group than in the TACE+RFA group (HR: 1.78; 95% CI: 1.15–2.75, *p* = 0.009, Fig. 2b). The OS was significantly better in the SR group than in the TACE group (HR: 3.17; 95% CI: 2.31–4.36, *p* < 0.0001, Fig. 2b). Moreover, the OS was significantly better in the TACE+RFA group than in the TACE group (HR: 1.82; 95% CI: 1.21–2.74, *p* = 0.004, Fig. 2b). The cumulative OS rates at 1, 3 and 5 years in the SR, TACE+RFA, and TACE groups were 89.2, 69.4 and 61.2%, 86.0, 57.9 and 38.2%, and 69.5, 37.0 and 15.2%, respectively (Fig. 2b).

### Baseline demographic data after propensity score matching

The SR group showed significant differences compared with the TACE+RFA and TACE groups with respect to baseline features before PSM. The SR group had significantly lower rate of HCV infection cirrhosis, tumor number, and mortality, lower serum total bilirubin and INR level, and higher rate of CP class A and tumor size compared to the TACE+RFA and TACE groups (*p* < 0.05) (Table 1). The PSM was performed with sex, age, cirrhosis, CP class, tumor size, and tumor number, and there were no significant differences for the important features (Tables 2 and 3).

**Table 2:** Comparison of surgical resection versus transarterial chemoembolization with or without radiofrequency ablation of patients withBCLC stage B hepatocellular carcinoma after propensity score matching

Variable	SR (*n* = 140)	TACE+RFA (*n* = 16)	P-value	SR (*n* = 140)	TACE (*n* = 87)	*P*-value
Male	117 (83.6)	13 (81.3)	0.220	117 (83.6)	67 (77.0)	0.813
Age (years)	62 (35–82)	66 (35–87)	0.249	62 (35–82)	64 (36–87)	0.121
Smoking	68 (48.6)	8 (50.0)	0.472	68 (48.6)	38 (43.7)	0.914
Alcohol use	58 (41.4)	7 (43.8)	0.858	58 (41.4)	35 (40.2)	0.858
HBV positive	70 (50.0)	10 (62.5)	0.206	70 (50.0)	36 (41.4)	0.343
HCV positive	30 (21.4)	6 (16.7)	0.069	30 (21.4)	26 (29.8)	0.148
Total Bilirubin	1.03 ± 0.43	1.21 ± 0.54	0.186	1.03 ± 0.43	1.11 ± 0.50	0.061
INR	1.00 ± 0.06	1.04 ± 0.10	0.061	1.00 ± 0.06	1.03 ± 0.09	0.051
Cirrhosis	36 (25.7)	6 (37.5)	0.098	36 (25.7)	31 (35.6)	0.111
Child-Pugh class A	134 (95.7)	14 (87.5)	0.236	134 (95.7)	80 (92.7)	0.158
Tumor size	8.2 ± 3.3	6.6 ± 2.8	0.903	8.2 ± 3.3	8.2 ± 3.5	0.063
Tumor size≥5 cm	127 (90.7)	12 (75.0)	0.186	127 (90.7)	75 (86.2)	0.071
Tumor number (≥3)	49 (35.0)	9 (56.2)	0.075	49 (35.0)	41 (47.1)	0.058
AFP (ng/mL) ≥ 200	34 (24.3)	2 (12.5)	0.405	34 (24.3)	17 (19.5)	0.289
Mortality	50 (35.7)	11 (68.8)	< 0.0001	50 (35.7)	70 (80.5)	0.010
Follow up times (months)	39 (1–98)	26 (9–76)	< 0.0001	39 (1–98)	21 (2–97)	0.240

*BCLC stage* Barcelona clinic liver cancer; *SR* Surgical resection; *TACE* Transcatheter arterial chemoembolization; *RFA* Radiofrequency ablation; *HBV* Hepatitis B virus; *HCV* Hepatitis C virus; *AFP: INR* International normalize ratio; Alpha-fetoprotein;

**Table 3:** Comparison of transarterial chemoembolization with radiofrequency ablation versus transarterial chemoembolization of patients with BCLC stage B hepatocellular carcinoma after propensity score matching

Variable	TACE+RFA (*n* = 56)	TACE (*n* = 231)	*P*-value
Male	42 (75.0)	173 (74.9)	0.987
Age (years)	64 (28–86)	64 (29–91)	0.672
Smoking	27 (47.4)	113 (48.9)	0.925
Alcohol use	23 (40.4)	100 (43.3)	0.763
HBV positive	32 (56.1)	103 (44.6)	0.091
HCV positive	21 (36.8)	90 (39.0)	0.085
Total Bilirubin	1.41 ± 0.67	1.34 ± 1.14	0.643
INR	1.10 ± 0.14	1.06 ± 0.12	0.060
Cirrhosis	43 (75.4)	155 (67.1)	0.160
Child-Pugh class A	43 (76.8)	194 (84.0)	0.203
Tumor size	5.5 ± 2.6	7.0 ± 3.8	0.062
Tumor size≥5 cm	25 (44.6)	149 (64.5)	0.053
Tumor number (≥3)	50 (89.3)	178 (77.1)	0.051
AFP (ng/mL) ≥ 200	6 (10.7)	50 (21.6)	0.064
Mortality	34 (59.6)	173 (74..9)	0.034
Follow up times (months)	36 (3–95)	22 (1–97)	< 0.0001

*BCLC stage*: Barcelona clinic liver cancer; *SR* Surgical resection; *TACE* Transcatheter arterial chemoembolization; *RFA* Radiofrequency ablation; *HBV* Hepatitis B virus; *HCV* Hepatitis C virus; *AFP: INR* International normalize ratio; Alpha-fetoprotein;

### Overall survival of patients in the different treatment groups after propensity score matching

**Fig. 3:**
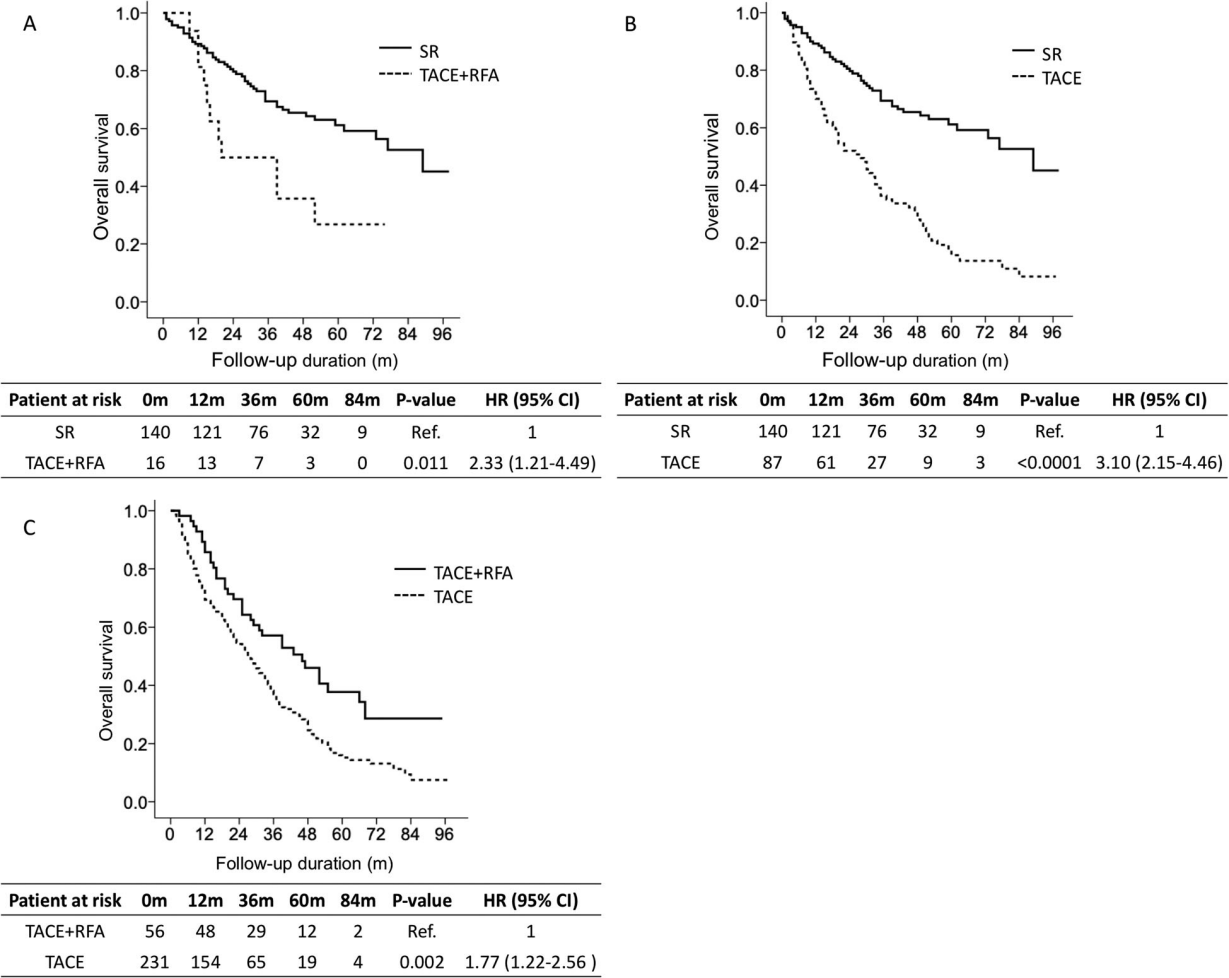
Overall survival according to different treatments after propensity score matching. Comparison of overall survival between surgical resection (SR) versus transarterial chemoembolization (TACE) with radiofrequency ablation (RFA) (**a**). Comparison of overall survival between SR versus TACE (**b**). Comparison of overall survival between TACE+RFA versus TACE (**c**)

In the SR group versus TACE+RFA group after PSM (Table 2), 140 patients underwent SR, and 16 patients received TACE+RFA. Patients undergoing SR had significantly higher survival rates than patients receiving TACE+RFA (HR: 2.33; 95% CI: 1.21–4.49, *p* = 0.011, Figs. 3a). The cumulative OS rates at 1, 3 and 5 years in the SR and TACE+RFA groups were 89.2, 69.4 and 61.2% and 81.3, 50.0 and 26.8%, respectively (Figs. 3a).

In the SR group versus TACE group after PSM (Table 2), 140 patients underwent SR, and 87 patients received TACE. Patients undergoing SR had significantly higher survival rates than patients receiving TACE treatments (HR: 3.10; 95% CI: 2.15–4.46, *p* < 0.0001, Figs. 3b). The cumulative OS rates at 1, 3 and 5 years in the SR and TACE groups were 89.2, 69.4 and 61.2% and 70.1, 36.3 and 15.7%, respectively (Figs. 3b).

In the TACE+RFA group versus TACE group after PSM (Table 3), 56 patients received TACE+RFA, and 231 patients received TACE. Patients undergoing TACE+RFA had significantly higher survival rates than patients receiving TACE treatments (HR: 1.77; 95% CI: 1.22–2.56, *p* = 0.002, Figs. 3c). The cumulative OS rates at 1, 3 and 5 years in the TACE+RFA and TACE groups were 85.7, 57.1 and 37.7% and 73.5, 37.0 and 15.2%, respectively (Figs. 3c).

## Discussion

Patients with BCLC stage B are recommended to receive TACE based on the BCLC system [2–4]. Our study showed that the SR group had higher OS than the TACE+RFA and TACE groups in BCLC stage B. Furthermore, the TACE+RFA group had higher OS than the TACE group. After PSM, the SR group still had higher OS than the TACE+RFA and TACE groups. In addition, the TACE+RFA group also had higher OS than the TACE group. SR should be considered a recommended treatment for select HCC patients in BCLC stage B.

TACE is recommended as a standard of care for the treatment of patients with BCLC stage B disease [2–4]. Several HCC experts have proposed four substages based on the Eastern Cooperative Oncology Group performance, CP class, and “up-to-7” criteria within BCLC stage B disease [11]. However, these criteria mostly indicate benefits from TACE. Based on the great improvements in surgical techniques and perioperative care, some treatments may not be suitable for patients with BCLC stage B HCC. Our results showed that SR resulted in a significantly higher OS rate than TACE+RFA and TACE in patients with BCLC stage B disease. Similarly, several studies from both Western and Eastern countries have demonstrated that SR results had higher long-term survival than nonsurgical treatments, even for patients with multiple tumors [6, 7, 12–14]. Furthermore, compared with TACE, SR significantly increases survival in select patients with BCLC stage B HCC [7]. Therefore, SR is a safe and effective therapy for select patients with resectable single or multiple HCC lesions and preserved liver function. Hence, SR may be recommended for select patients with BCLC stage B disease.

A previous study showed that TACE+RFA is safe and as effective as SR for patients with HCC within the Milan criteria and BCLC stage B [6, 10]. Our study demonstrated that the SR group had a higher OS than the TACE+RFA group, although the SR group had larger tumor sizes but fewer tumor numbers than the TACE+RFA group. After PSM with sex, age, tumor size, tumor number, cirrhosis, and CP class, the SR group still had higher OS than the TACE+RFA group. Our study first demonstrated that SR has a significantly higher OS than TACE+RFA in the literature. Indeed, SR may be considered for select patients who fit these criteria and could be recommended for patients with BCLC stage B disease.

Our study showed that the TACE+RFA group had a higher OS than the TACE group, although the TACE+RFA group had smaller tumor sizes and more tumor numbers than the TACE group. After PSM, the TACE+RFA group still had a higher OS than the TACE group. Our study is consistent with previous studies showing that TACE+RFA has a better OS than TACE in BCLC stage B [6, 8, 9]. Hence, combination TACE and RFA treatment may be considered for select patients who were multiple tumors with smaller tumor sizes and could be recommended for patients with BCLC stage B disease.

Our study has several limitations. First, we did not take into consideration comorbidity and antiviral therapy on OS. Second, we did not consider the possible differences in TACE cycles. Third, as with all retrospective studies, there was some selection bias despite our use of PSM. Furthermore, a randomized study between the different treatments will be performed.

## Conclusions

The SR group had higher OS than the TACE+RFA and TACE groups. Furthermore, the TACE+RFA group had higher OS than the TACE group in BCLC stage B.

## Data Availability

Data is available from the corresponding author upon reasonable request.

## Abbreviations

HCC: Hepatocellular carcinoma
BCLC: Barcelona Clinic Liver Cancer
AASLD: American Association for the Study of Liver Disease
EASL: European Association for the Study of Liver
APASL: Asian-Pacific Associated for the Study of the Liver
SR: Surgical resection
RFA: Radiofrequency ablation
TACE: Transcatheter arterial chemoembolization
PSM: Propensity score matching
CP class: Child-Pugh class
OS: overall survival
HBV: Hepatitis B virus
HCV: Hepatitis C virus
AFP: Alpha-fetoprotein
INR: International normalize ratio
CT: Computed tomography
MRI: Magnetic resonance imaging
HR: Hazard ratio
CI: Confidence interval
